# Advances in Plasma Treatment of Materials

**DOI:** 10.3390/ma18245494

**Published:** 2025-12-06

**Authors:** Mirosław Dors

**Affiliations:** Institute of Fluid Flow Machinery, Polish Academy of Sciences, 80231 Gdansk, Poland; mdors@imp.gda.pl

## 1. Introduction

Plasma technologies have evolved from niche applications into a cornerstone of modern material engineering. Operating under non-equilibrium conditions, cold atmospheric plasmas, low-pressure plasmas, and dielectric barrier discharges (DBDs) enable modification of surface properties without affecting the bulk characteristics of materials. This Special Issue, “Advances in Plasma Treatment of Materials,” features six cutting-edge studies that showcase the versatility of plasma processing. The collection spans diverse fields, including the synthesis of silicon oxycarbide thin films, the surface functionalization of biodegradable polymers, environmental remediation, energy conversion, and advanced metallurgical finishing.

## 2. Advanced Thin-Film Synthesis and Mechanisms

Precise control of the chemical structure during deposition is the holy grail of materials science. In this issue, Walkiewicz-Pietrzykowska et al. [1] provide a breakthrough in the synthesis of silicon oxycarbide (SiOC:H) thin films. Utilizing Remote Hydrogen Plasma Chemical Vapor Deposition (RHP-CVD), the authors successfully converted a cyclic organosilicon precursor (octamethyl-1,4-dioxatetrasilacyclohexane) and its polymer equivalent (POBDMS) into high-quality films.

This study elucidates a selective mechanism where hydrogen radicals specifically cleave Si-Si bonds while preserving the Si-C, C-H, and Si-O skeleton. This process allows for tunable transitions from polymer-like to ceramic-like structures based simply on substrate temperature, providing a pathway for creating dense, high-refractive-index coatings for optical and protective applications.

## 3. Surface Engineering for Biomedicine and Biodegradable Polymers

Plasma treatment is particularly valuable for modulating the interface between materials and biological environments. Two papers in this issue address the critical need for sterilization and surface activation.

Mazur-Lesz et al. [2] investigate the application of cold atmospheric plasma (CAP) in dentistry to combat denture stomatitis. Their study on prosthetic materials reveals that a 10-min helium/oxygen plasma treatment significantly reduces the adhesion of *Candida albicans* and *Candida glabrata*. While acetal and metal alloys remained morphologically stable, acrylic resins showed increased roughness and color changes, suggesting that material-specific plasma protocols are essential for clinical adoption.

In the realm of sustainable packaging, Stepczyńska and Śpionek [3] explore the modification of Thermoplastic Starch (TPS) films using both corona discharge and low-pressure plasma. TPS films, while attractive for sustainable packaging, suffer from low surface energy and limited durability. The authors demonstrate that corona and low-pressure plasmas significantly increase surface polarity, O/C ratios, wettability, and adhesion by introducing polar functional groups and inducing controlled ablation. The treatment also offers sterilization potential, positioning plasma as a multifunctional tool for improving the performance of biodegradable packaging. The work concludes by outlining critical next steps, including in vivo biocompatibility testing to evaluate potential biomedical uses, such as wound dressings or drug delivery systems.

## 4. Environmental Remediation and Energy Conversion

Plasma technologies are increasingly pivotal in addressing global sustainability challenges ranging from food safety to greenhouse gas utilization.

Recek et al. [4] present a robust kinetic model for the decontamination of food and feedstock. They utilized low-pressure oxygen plasma to degrade aflatoxins, highly toxic carcinogens found on agricultural products. Their study distinguishes between thermal effects and plasma kinetics, demonstrating that the degradation is driven by atomic oxygen flux. A dose of approximately 10^25^ m^−2^ was sufficient to reduce toxin concentrations below detection limits, with H-mode plasma achieving this in mere seconds, offering a scalable solution for food safety.

Addressing the climate crisis, Dorosz et al. [5] tackle the conversion of CO_2_ into CO using a Dielectric Barrier Discharge (DBD) reactor. This work focuses on the synergy between plasma and packing materials (plasma catalysis). The authors introduce a “mass-normalized conversion efficiency factor” to evaluate economic viability. While corundum Al_2_O_3_ showed the highest intrinsic activity, kaolin emerged as a cost-effective alternative that induces turbulence and enhances discharge uniformity, offering a practical route for scaling up CO_2_ utilization technologies.

## 5. Sustainable Metallurgical Finishing

Finally, this issue also addresses the finishing of metallic surfaces. Pérez-Durán et al. [6] introduce an eco-friendly approach to the Plasma Electrolytic Polishing (PEP) of AISI 304 stainless steel. Replacing toxic acid electrolytes with an aqueous urea/ammonium nitrate solution, the process reduced surface roughness by 54% and decreased the corrosion rate by 99%. XPS analysis confirmed the formation of a protective passive layer enriched with Cr_2_O_3_ and Fe_3_O_4_, proving that plasma polishing can simultaneously enhance aesthetics and durability via green chemistry.

## 6. Conclusions

The research presented in this Special Issue underscores the transformative power of plasma treatment. Whether it is chemically engineering the backbone of a precursor for ceramics, sterilizing delicate biomaterials, detoxifying food supplies, converting waste gases, or polishing metals, plasma provides a tunable, efficient, and increasingly sustainable toolkit. I hope these contributions inspire further interdisciplinary research to push the boundaries of what is possible with ionized gases.

## References

[1] Walkiewicz-Pietrzykowska A., Jankowski K., Kurjata J., Dolot R., Brzozowski R., Zakrzewska J., Uznanski P. (2025). Silicon Oxycarbide Thin Films Produced by Hydrogen-Induced CVD Process from Cyclic Dioxa-Tetrasilacyclohexane. Materials.

[2] Mazur-Lesz A., Pawłat J., Terebun P., Zarzeczny D., Grządka E., Starek-Wójcicka A., Kwiatkowski M., Malinowska I., Mnichowska-Polanowska M., Machoy M. (2025). Cold-Plasma Method in Counteracting Prosthetic Stomatitis: Analysis of the Influence of Cold Plasma on Prosthetic Materials. Materials.

[3] Stepczyńska M., Śpionek A. (2025). Plasma Modification Effects of Thermoplastic Starch (TPS) Surface Layer: Film Wettability and Sterilization. Materials.

[4] Recek N., Zaplotnik R., Primc G., Gselman P., Mozetič M. (2025). Low-Temperature Degradation of Aflatoxins via Oxygen Plasma: Kinetics and Mechanism Driven by Atomic Oxygen Flux. Materials.

[5] Dorosz A., Zaraska K., Lewak M., Małolepszy A., Jaworski J., Moskal A. (2025). The Use of Metal Oxides (Al_2_O_3_ and ZrO_2_) and Supports (Glass and Kaolin) to Enhance DBD Plasma-Catalytic CO_2_ Conversion. Materials.

[6] Pérez-Durán H., Martínez-Baltodano F., Vargas-Gutiérrez G. (2025). Polishing of AISI 304 SS by Electrolytic Plasma in Aqueous Urea Solution: Effect on Surface Modification and Corrosion Resistance. Materials.

